# sTREM-1 and TNF-α levels are associated with the clinical outcome of leprosy patients

**DOI:** 10.3389/fmed.2023.1177375

**Published:** 2023-06-29

**Authors:** Márcio Bezerra-Santos, Lays G. Santos Bomfim, Camilla N. Oliveira Santos, Maria Wiliane N. Cunha, Eduardo J. Rocha de Moraes, Rodrigo A. Cazzaniga, Martha D. L. Tenório, Jonnia M. Sherlock Araujo, Lucas Menezes-Silva, Lucas Sousa Magalhães, Aline S. Barreto, Steven G. Reed, Malcolm S. Duthie, Michael W. Lipscomb, Roque Pacheco de Almeida, Tatiana Rodrigues de Moura, Amélia Ribeiro de Jesus

**Affiliations:** ^1^Centro de Ciências Médicas e Enfermagem, Universidade Federal de Alagoas, Maceió, Brazil; ^2^Laboratório de Imunologia e Biologia Molecular, Universidade Federal de Sergipe, Aracaju, Brazil; ^3^Programa de Pós-Graduação em Ciências da Saúde, Universidade Federal de Sergipe, Aracaju, Brazil; ^4^Departamento de Dermatologia, Hospital Universitário, Universidade Federal de Sergipe, Aracaju, Brazil; ^5^Host Directed Therapeutics (HDT) Bio Corp, Seattle, WA, United States; ^6^Department of Pharmacology, University of Minnesota, Minneapolis, MN, United States; ^7^Departamento de Medicina, Universidade Federal de Sergipe, Aracaju, Brazil; ^8^Instituto de Investigação em Imunologia (III), Institutos Nacionais de Ciência e Tecnologia (INCTs), Conselho Nacional de Pesquisa e Tecnologia (CNPq), São Paulo, Brazil

**Keywords:** leprosy, soluble TREM-1, inflammatory cytokine, immune markers, leprosy complications

## Abstract

Leprosy reaction (LR) and physical disability (PD) are the most significant clinical complications of leprosy. Herein, we assessed the circulating serum-sTREM-1 and TNF-α levels and their genetic polymorphisms in leprosy. Serum-sTREM-1 and TNF-α levels were measured in leprosy patients (LP) before treatment (*n* = 51) and from their household contacts (HHCs; *n* = 25). DNA samples were genotyped using TREM-1 rs2234246 and TNF-α rs1800629-SNP in 210 LPs and 168 endemic controls. The circulating sTREM-1 and TNF-α levels are higher in the multibacillary form. The ROC curve of the serum-sTREM-1 levels was able to differentiate LR from non-LR and PD from non-PD. Similarly, LPs with serum-sTREM-1 levels >210 pg/ml have 3-fold and 6-fold higher chances of presenting with LR and PD, respectively. Genotypes CC+CT of the TREM-1 were associated with leprosy. Taken together, our analyses indicated that sTREM-1 and TNF-α play an important role in the pathogenesis of leprosy and provide promising biomarkers to assist in the diagnosis of leprosy complications.

## Introduction

Leprosy is a chronic infectious disease caused by the intracellular bacillus *Mycobacterium leprae* and *Mycobacterium lepromatosis* (1). Regardless of the significant reduction in prevalence after the widespread use of multidrug therapy, the new case detection rates have stabilized in the last few years, and leprosy remains endemic in a number of localized regions, such as Brazil, India, and China (2). Note that leprosy reaction (LR) and the occurrence of physical disability (PD) are the most important clinical complications of leprosy (3).

In this regard, several studies have reported the influence of the immunological response on leprosy infection. T helper 1 (Th1) and Th17 cell responses are associated with the control of *M. leprae* while exacerbating the Th1 response, and a high number of CD8^+^ T cells may be involved with increased disease severity. Alternatively, Th2 and Treg cells are related to the multibacillary presentation, with largely infected macrophages in skin lesions (4).

The triggering receptor expressed on myeloid cells-1 (TREM-1) is a cell-surface receptor constitutively expressed mainly on neutrophils and monocytes. This receptor is involved in the amplification of the inflammatory response by activating transcription factors such as NF-κB (5). Beyond the membrane form (mbTREM-1), TREM-1 can also be found in a soluble form (sTREM-1), which acts mainly negatively, modulating mbTREM-1 receptor signaling (6). Regardless of whether the cellular source of sTREM-1 remains unclear, the role of sTREM-1 related to some infectious diseases has been largely investigated, including several reports showing that sTREM-1 is directly associated with severe disease, as in visceral leishmaniasis (7), pulmonary tuberculosis (8), sepsis (9), and COVID-19 (10).

The TREM-1 gene is present on chromosome 6, and a polymorphism (rs2234246 SNP) has been reported in non-disease individuals to affect the sTREM-1 levels and the expression of messenger RNA to the mbTREM-1. Moreover, the minor allele T was associated with the increased production of this protein (11). Although several genetic studies have been published on leprosy, no studies have been reported on this TREM-1 polymorphism (12–14).

Importantly, the clinical signs of leprosy may be scarce in the early stages of the disease, leading to delayed diagnosis or misdiagnosis (15). Furthermore, patients search for medical support when presenting with some clinical complications (16). Thereby, the identification of some biomarkers to help with the early diagnosis of leprosy and its clinical complications is urgently required. Herein, we reported that circulating sTREM-1 and TNF-α are related to the lepromatous leprosy (LL) form, especially the LR and PD. Thus, these molecules might be promising biomarkers to monitor the occurrence of LR and PD during the clinical follow-up of leprosy treatment.

## Materials and methods

### Ethics statement

This project was approved by the Ethics and Research Committee of the Federal University of Sergipe (CAAE 0152.0.107.000-07). All subjects or their legal representatives signed a free and clarified term of knowledge contract (IC) agreeing to participate in the study.

### Study subjects and data collection

This is a case-control study with two different approaches: first, a case-control study of sera measurements, including 51 leprosy patients and 25 controls recruited from January 2019 to December 2019. Second, a case-control study of genetic markers, including 210 leprosy patients and 168 controls who were enrolled in the study between January 2010 and December 2019.

All leprosy patients included in this study attended the dermatology clinic of the Hospital Universitário at the Universidade Federal de Sergipe, Aracaju City, northeastern Brazil. Leprosy patients were completely examined by dermatologists, and the inclusion criteria were to have a confirmed diagnosis of leprosy prior to starting treatment with conventional multidrug therapy (MDT). In accordance with the Brazilian Ministry of Health, patients were diagnosed by clinical evaluation (dermato-neurological) and histopathological and lymph bacilloscopic examinations (17). Additionally, for the purpose of treatment classification, leprosy patients were classified according to their operational forms: paucibacillary (PB), if they exhibited fewer than five skin lesions and received a negative bacilloscopic examination; or multibacillary (MB), if they presented with five or more skin lesions and tested positive on the bacilloscopic examination. To determine their clinical forms, histopathological examinations of skin biopsies were performed and classified according to Ridley-Jopling's criteria (18) as follows: indeterminate leprosy (IL), tuberculoid leprosy (TT), borderline leprosy (BL), or lepromatous leprosy (LL).

All patients who were invited and included in the study were recruited through convenience sampling at the time of diagnosis and consecutive order. The exclusion criteria were applied to individuals who had diseases known to affect the immune response or that confound the diagnosis of leprosy complications, such as HIV and HTLV-I infections, diabetes, or neurological diseases. The selection of patients did not consider factors such as sex and age as criteria, but efforts were made to match groups to prevent any bias during the analysis.

The control group used in the analysis of serum-sTREM-1 and TNF-α levels was composed of household contacts (HHCs), including any person living in close and prolonged contact with the leprosy patients but not genetically related. These contacts most commonly were the patient's spouses. Moreover, we only included patients who had started MDT. These patients were followed-up monthly during treatment to detect symptoms of LR and neurological disabilities, following the recommendations of the World Health Organization (19), using a specific questionnaire and the neurological simplified evaluation (but this information was not included in this study).

For the genetic analysis, the controls in the study included HHC and an additional population of 115 unrelated individuals living in the same city as the patients, which is an endemic area for leprosy. These two groups were combined to form the “endemic control” group (EC, *n* = 154) in the analyses, representing the control sample. However, owing to a lack of available information and low DNA concentrations, some subjects were excluded, resulting in potential variations in the total number of individuals included in the SNP analysis across the results.

### Measurement of serum-strem-1 and TNF-α levels

Sera were obtained from whole blood collected from the leprosy patients before treatment and from HHCs. Serum-sTREM-1 levels were assessed using specific enzyme-linked immunosorbent assay (ELISA) kits (DuoSet-R&D Systems, Abingdon, UK) using the manufacturer's recommended protocol and measured using a microplate reader (Epoch-BioTek, Luzern, Switzerland). A standard curve was generated for each set of samples assayed. Concentrations of the cytokine TNF-α were determined using multiplex assay, according to the manufacturer's instructions, by using a MILLIPLEX– Human Th17 Magnetic Bead Panel kit (Merck Millipore Corporation, USA).

### Genotyping TREM-1 rs2234246 and TNF-α rs1800629 SNP

Genomic DNA was extracted from blood samples using the PureLink^®^ Genomic-DNA Kit (Invitrogen™, USA). The concentration and purity of DNA were quantified using NanoDrop™ (Thermo-Scientific™, USA). We genotyped DNA samples using commercial TREM-1 rs2234246 and TNF-α rs1800629 TaqMan^®^ probes (Applied Biosystems™, USA) and TaqMan™ Genotyping Master Mix (Applied Biosystems™, USA) by qPCR, using 7,500 Real-Time PCR (Applied Biosystems™) following the manufacturer's instructions. The results were assessed using TaqMan^®^ Genotyper software version 1.6.0. Information about the analyzed SNPs and assay codes for each probe are presented in Supplementary Table 1.

### Statistical analysis

The clinical and demographical data, as well as serum-sTREM-1 and TNF-α levels, were compared across subgroups according to the operational (PB, MB, and HHCs) and clinical forms (IL, TT, BL, and LL) of leprosy and clinical complications (LR or PD). The mean, median, and standard deviation of the groups were calculated. The receiver operating characteristic curve (ROC curve) was used to distinguish groups based on the levels of sera measurements.

D'Agostino–Pearson normality tests were applied to verify if the data exhibited Gaussian distributions. Statistical differences between the groups were determined by the Mann–Whitney U test for two groups or the Kruskal–Wallis test for more groups, followed by the Dunn test for multiple comparisons. Correlations between the cytokine levels were determined using the Spearman correlation test.

For genetic polymorphism analyses, the allelic and genotype frequencies were compared according to the operational and clinical forms of leprosy, and the odds ratio (OR) was calculated using Fisher's exact or the Chi-squared test. The Hard-Weinberg equilibrium (HWE) test was performed using GENEPOP Online 4.2 (20).

All analyses were performed using GraphPad Prism software 8.0.1 (GraphPad Software Inc., USA). To evaluate differences, alpha (∂) was set at 5%, and tests were made using a two-tailed *p-value*.

## Results

### Clinical and demographic characteristics of subjects

No differences were identified among the mean ages between PB and MB patients or HHCs (Table 1). Nonetheless, the proportion of men presenting with the MB form (57.1%) was significantly higher than the PB form (26.1%; OR = 3.77; *p* = 0.02). Remarkably, the occurrence of LR was significantly higher among MB (71.4%) than PB (17.4%; OR = 11.88; *p* < 0.001). Similarly, patients presenting with a PD degree of 1 or 2 were higher in the MB (60.7%) than in the PB group (10.7%; OR = 3.53; *p* = 0.03).

**Table 1:** Demographic and clinical characteristics of patients and household contacts.

**Variables**	**MB (*n* = 28)**	**PB (*n* = 23)**	**HHC (*n* = 25)**	**OR**	**95% CI**	***p-*value**
**Age**						
Variation	10–68	11–81	20–72	–	–	^*^0.22
Mean ± SD	43.6 ± 15.9	49.6 ± 18.9	46.9 ± 14.5			
**Men** *n* (%)	16 (57.1%)	06 (26.1%)	09 (39.1%)	3.77	1.14 to 12.48	^**^0.02
**Number of lesions**						
Variation	2–14	1–5	–	–	–	^*^ < 0.0001
Mean ± SD	6.58 ± 2.85	1.91 ± 1.59	–			
**Leprosy reaction** *n* (%)	20 (71.4%)	4 (17.4%)	–	11.88	1.53 to 5.16	^**^0.0001
**Physical disability degree** ***n*** **(%)**						
Degree 0	11 (39.3%)	16 (50%)	–	3.53	1.09 to 11.36	^**^0.03
Degree 1 or 2	17 (60.7%)	7 (10.7%)	–			

MB, Multibacillary; PB, Paucibacillary; HHCs, Household contacts; OR, odds ratio; CI, Confidence interval; SD, Standard deviation. ^*^Unpaired test; ^**^Fisher exact test. OR + 95% CI refers to MB × PB. All data were obtained before treatment.

### Serum-sTREM-1 levels are higher in the MB form and in patients presenting with leprosy reactions and physical disability

**Figure 1:**
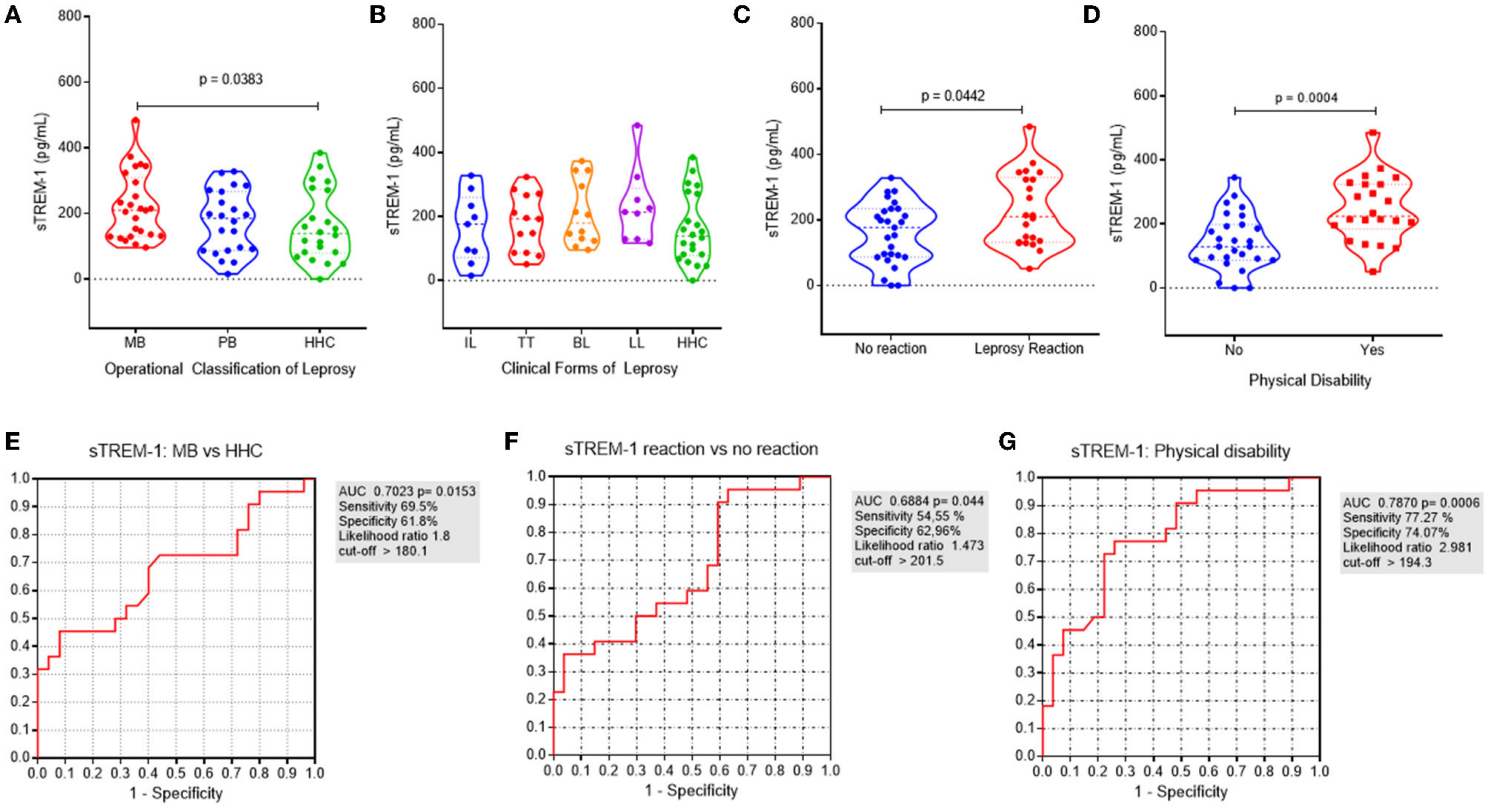
sTREM-1 serum levels are associated with MB presentation and clinical complications on leprosy. **(A)** sTREM-1 serum levels according to the operational classification on leprosy: multibacillary (MB-in red), paucibacillary (PB-in blue), and household contacts (HHCs-in green). **(B)** sTREM-1 serum levels according to the clinical forms of leprosy: indeterminate (IL–in blue), tuberculoid (TT–in red), borderline (BL–in orange), lepromatous (LL–in violet), and HHCs (in green). **(C)** sTREM-1 serum levels according to the occurrence of leprosy reaction (LR): no reaction (in blue) and LR (in red). **(D)** sTREM-1 serum levels according to the occurrence of physical disability (PD): no PD (in blue) and with PD (in red). The receiver operating characteristic (ROC) curve was generated to discriminate the levels of sTREM-1 between **(E)** leprosy patients presenting with the MB operational form and HHCs [area under the ROC curve (AUC) = 0.7023; *p* = 0.01]; **(F)** leprosy patients presenting with LR and those with no LR (AUC = 0.6884; *p* = 0.04); **(G)** leprosy patients presenting with some PD (degrees 1 or 2) and those with no PD-degree 0 (AUC = 0.787; *p* = 0.0006).

We observed higher levels of sTREM-1 in MB patients (221 ± 102.2 pg/ml) than HHCs (160.9 ± 107.5 pg/ml; *p* = 0.03; Figure 1A), and no differences were found when comparing all leprosy patients with HHCs (198.9 ± 100.7 pg/ml; *p* = 0.15) or among patients when compared by the Ridley-Jopling classification (Figure 1B). Correspondingly, we observed higher levels of sTREM-1 in patients presenting with LR (228.5 ± 112.1 pg/ml) and PD (247.9 ± 101 pg/ml) than those with no clinical complications (160.1 ± 92.4 pg/ml; *p* = 0.04 and 144.3 ± 87.3 pg/ml; *p* < 0.001, respectively; Figures 1C, D). However, the correlation analysis using the patient classification according to the number of lesions (PB and MB) and sTREM-1 levels resulted in a poor and insignificant result (*r* = 0.178, *p* = 0.21) (Supplementary Figure 1A).

Additionally, using the receiver operating characteristic (ROC) curve, serum-sTREM-1 levels had 69.5% sensitivity and 61.8% specificity for differentiating MB patients from those with HHCs [area under the ROC curve (AUC) = 0.7023; *p* = 0.01; Figure 1E]. Furthermore, the ROC curve of serum-sTREM-1 levels had 54.55% sensitivity and 62.96% specificity for differentiating LR from non-LR patients (AUC = 0.6884; *p* = 0.04; Figure 1F). More importantly, sTREM-1 levels had 77.27% sensitivity and 74.07% specificity for differentiating PD from non-PD patients (AUC = 0.787; *p* < 0.001; Figure 1G).

Considering that serum-sTREM-1 levels increased in patients presenting with MB, LL, LR, and LD, we grouped them into those with serum-sTREM-1 levels > 210 pg/ml and <210 pg/ml. Thereafter, we compared the clinical characteristics among those groups (Supplementary Table 2). We used the value of sTREM-1 > 210 pg/ml, as it presented the highest sensitivity and specificity rates for the most severe leprosy outcomes. Interestingly, patients with serum-sTREM-1 levels >210 pg/ml had almost 5-fold higher odds of presenting with the LL form (OR = 4.81; *p* = 0.04). Similarly, leprosy patients have 3-fold higher odds of presenting with LR (OR = 2.81; *p* = 0.06) and 6-fold higher odds of presenting with physical disability (OR = 5.83; *p* = 0.004).

### Serum-TNF-α levels are higher in MB and LL clinical form

We identified elevated levels of TNF-α in MB (363.2 ± 105.6 pg/ml) than PB patients (300.7 ± 79.1 pg/ml; *p* = 0.03) and HHCs (296.1 ± 60.2 pg/ml; *p* = 0.01; Supplementary Figure 2A), while a comparison of all leprosy patients showed higher but no-significant levels of TNF-α than control patients (334.5 ± 98.6 pg/ml; *p* = 0.08). Similarly, LL patients (427 ± 119.1 pg/ml) also presented higher levels of TNF-α compared to IL patients (304 ± 85.5 pg/ml; *p* = 0.03), TT patients (298.6 ± 78 pg/ml; *p* = 0.01), and HHCs (*p* = 0.002; Supplementary Figure 2B). No differences were observed according to the occurrence of LR or PD (Supplementary Figures 2C, D). In addition, the ROC curve of serum-TNF-α levels had 91.3% sensitivity and 77.8% specificity for differentiating MB patients from HHCs and PB (Supplementary Figures 2E, F). Correspondingly, the ROC curve of serum-TNF-α levels had 91.3% sensitivity and 77.78% specificity for differentiating the LL form from HHCs and IL+TT (Supplementary Figures 2G, H). Complementarily, the correlation analysis among TNF-a levels and the operational classification of patients showed a poor but significant correlation *r* = 0.319 (*p* = *0*.024) (Supplementary Figure 1B).

**Figure 2:**
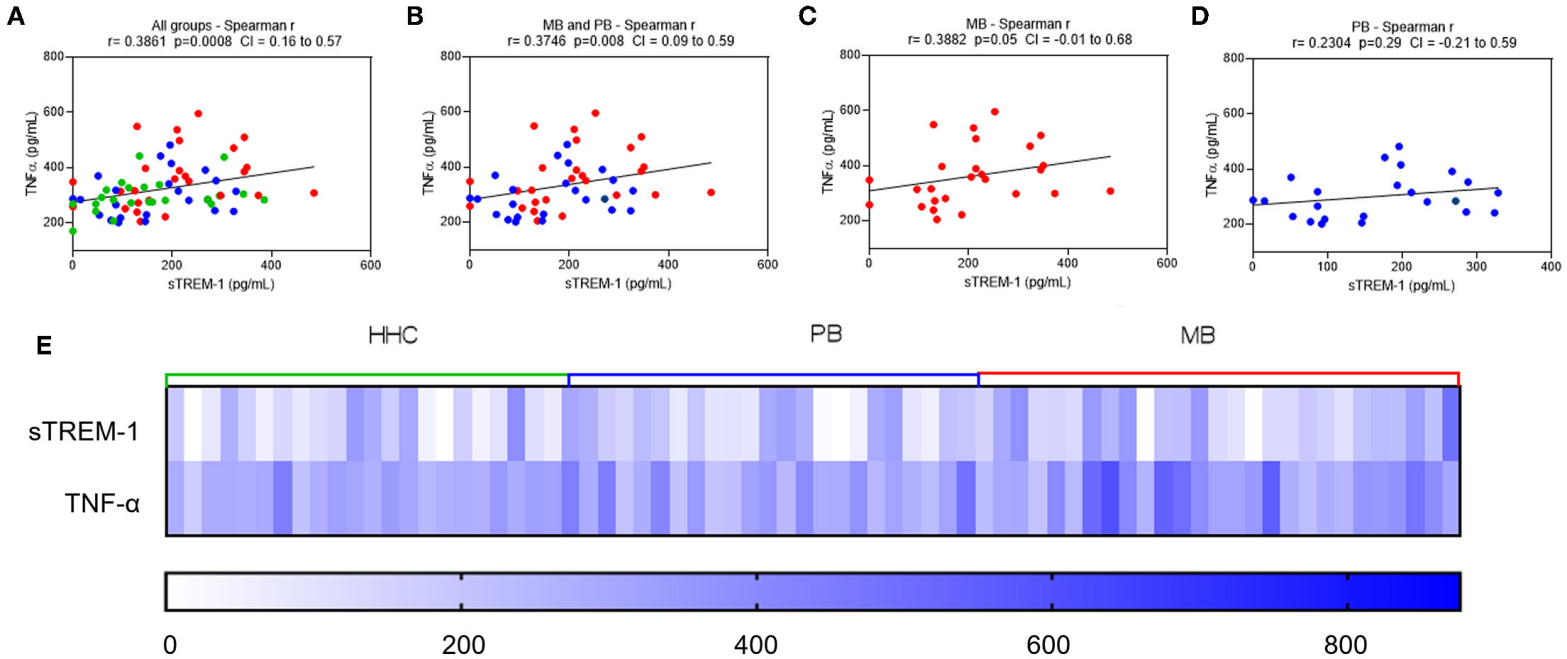
Serum levels of sTREM-1 are correlated to the TNF-α. Correlations between serum levels of TNF-α and sTREM-1 in leprosy patients (multibacillary–MB, in red; paucibacillary–PB, in blue) and household contacts (HHC–in green): **(A)** all groups (MB, PB, and HHC); **(B)** MP and PB groups; **(C)** MB group; and **(D)** PB group. Dotted lines on the x-axis represent the values of sTREM-1. Dotted lines on y-axis represent the values of TNF-α. Correlations were analyzed using the Spearman test. **(E)** A heatmap expressing the serum levels of sTREM-1 and TNF-α in MB and PB leprosy patients and HHCs.

Considering the higher expression of sTREM-1 and TNF-α in MB forms, we performed the Spearman correlation (Figures 2A–D). Interestingly, we observed a weak but significant correlation among sTREM-1 and TNF-α in all groups (Rho = 0.3861; *p* < 0.001; Figure 2A), among PB and MB (Rho = 0.3742; *p*-value = 0.008; Figure 2B), and only MB (Rho = 0.38; *p* = 0.05; Figure 2C). Similarly, the heatmap showed higher expression of sTREM-1 and TNF-α in the MB forms (Figure 2E).

### Association between the TREM-1 rs2234246 and TNF-α rs1800629 SNPs with the occurrence of leprosy

Considering the results in the serum expression of sTREM-1 and TNF-α, we decided to genotype the study population for the TREM-1 rs2234246 and TNF-α rs1800629 SNP and verify whether the differences found could be related to genetic polymorphisms. The characteristics of subjects according to the groups and the leprosy clinical forms are shown in Supplementary Table 3. The frequency of males is higher in leprosy patients than in EC. No deviation was found in HWE. There was a higher frequency of CC+CT genotypes of the TREM-1 rs2234246 than the TT genotype in leprosy patients (OR = 1.598; *p* = 0.04; Table 2). Nevertheless, there were no differences between the allele or genotype frequencies of TREM-1 and TNF-α SNPs, considering the clinical outcomes of leprosy (Supplementary Table 4). Additionally, no differences were observed when the alleles of these SNPs were compared according to the Ridley and Jopling classification (Supplementary Table 5).

**Table 2:** Frequency and distribution of TREM-1 rs2234246 in leprosy patients and the control group.

	**Allele and genotype frequencies** ***n*** **(%)**^**a**^						
		**Case group**	**Control group**				
**TREM-1 rs2234246**		**LP**	**HHC**	**Allele/Genotype**	**OR**	**95% CI**	***p-*** **value** *^b^*
		***n*** = **190**	***n*** = **39**				
	C	170 (44.7)	33 (42.3)	C vs. T	1.10	0.67–1.81	0.71
	T	210 (55.3)	45 (57.7)				
	CC	44 (23.2)	6 (15.4)				
	CT	82 (43.1)	21 (53.8)	CC + CT vs. TT	0.87	0.41–1.82	0.85
	TT	64 (33.7)	12 (30.8)				
		**LP**	**EC**				
		***n*** = **190**	***n*** = **154**				
	C	170 (44.7)	119 (38.6)	C vs. T	0.77	0.57–1.05	0.12
	T	210 (55.3)	189 (61.4)				
	CC	44 (23.2)	34 (22.1)				
	CT	82 (43.1)	51 (33.1)	CC + CT vs. TT	1.59	1.02–2.45	**0.04**
	TT	64 (33.7)	69 (44.8)				

LP, leprosy patients; HHC, household contacts; EC, endemic control; OR, odds ratio; CI, confidence interval.

a (%) percentual of the subjects with the specified allele or genotype.

b Test for association was performed using the Fisher's exact test. Bold indicate statistically significant results.

Conversely, we observed an association between the presence of a higher producer of sTREM-1 (sTREM-1 > 210 pg/ml) and the TT genotype (*p* = 0.03; Supplementary Table 6). In accordance with this, when we compared the amount of sTREM-1 according to genotypes for the SNP analyzed (TREM-1 rs2234246; Supplementary Figures 3A, B), including all case and control groups, higher quantities of sTREM-1 were detected in the TT genotype patients than in the CT genotype patients (*p* = 0.03).

## Discussion

Early identification of the clinical complications of leprosy has a major effect on the clinical management and outcome of these patients (21). Considering this, it is relevant to find new biomarkers to help identify those presenting with LR or PD. Our findings suggest that assessing the sTREM-1 in serum samples from leprosy patients may be a valuable new approach to assist in the diagnosis of LR and PD during the follow-up of these patients.

Herein, we observed higher levels of sTREM-1 in MB patients compared to HHCs. Similarly, higher levels of sTREM-1 were identified in the LL clinical form. Studies assessing sTREM-1 levels in other diseases caused by intracellular pathogens have demonstrated the value of this molecule in differentiating severe from non-severe forms of tuberculosis and leishmaniasis. Feng et al. demonstrated that serum-sTREM-1 levels are significantly increased in pulmonary tuberculosis and are correlated with more advanced involvement in chest x-rays and a higher bacteria burden in sputum (8). More importantly, higher levels of sTREM-1 are independent predictors of on-treatment mortality in tuberculosis.

Furthermore, in a meta-analysis by Wu et al. (22), sTREM-1 had a moderate diagnostic performance in differentiating sepsis from non-sepsis in adult patients. As a result, the authors indicated that a combination of several markers appears to be a useful approach to improving accuracy in diagnosing sepsis. Additionally, Gibot et al. (23), in a prospective study, demonstrated the high performance of a bioscore combining sTREM-1 along with procalcitonin and CD64 on neutrophils index in diagnosing sepsis.

Interestingly, we identified higher serum-sTREM-1 levels in leprosy patients with LR and PD. Additionally, leprosy patients with serum-sTREM-1 levels above 210 pg/ml have almost a 3-fold higher chance of presenting with LR and a 6-fold higher chance of presenting with PD. Notably, those clinical outcomes are the most severe complications of leprosy, and they are causally related to an exacerbated inflammatory response, nerve damage, lack of sensibility, and loss of life quality in leprosy patients (24). Few studies have investigated the role of TREM-1 in neural tissues, and most of them have focused on TREM-2 (25–28). However, previous studies have already demonstrated the role of TREM-1 in amplifying the inflammatory process and protein autophagy that are associated with tissue damage, as already observed in studies with Parkinson's and Alzheimer's diseases (25, 26). Regardless of these findings, the role of sTREM-1 in neural tissues remains unclear, and it is necessary to investigate whether Schwann cells are a source of sTREM-1, attracting and activating neutrophils. Therefore, we could hypothesize that high sTREM-1-serum levels in leprosy may indicate an inflammatory process that occurs in LR and neural damage during *M. leprae* infection. However, experimental and new clinical data are mandatory to confirm this.

Additionally, we identified higher serum levels of TNF-α in MB and LL clinical forms. In previous studies, TNF-α has been extensively described as an important proinflammatory cytokine associated with tissue damage in leprosy (29). Importantly, we have demonstrated that sTREM-1 is positively correlated with TNF-α, although there is a weak but significant correlation. Similarly, Liu et al. (30) confirmed that both serum contents of sTREM-1 and TNF-α are significantly increased in patients with mycoplasma pneumoniae infection. The authors indicated that TREM-1 overexpression enhances the nuclear translocation of NF-kβ and exerts a proinflammatory response, as evidenced by triggering TNF-α release. When exacerbated, the unregulated inflammatory response can lead to tissue damage, as it usually occurs in LR and as nerve impairment in patients presenting with PD (3, 21, 31).

Concerning the genetic polymorphism analysis, associations between the genotypes CC+CT for TREM-1 rs2234246 and leprosy *per se* or the occurrence of leprosy were observed. The TREM-1 rs2234246 SNP C/T is a functional polymorphism that has been tested in healthy individuals and reported to affect sTREM-1 levels and the expression of mbTREM-1. Furthermore, the T allele is associated with increased levels of this molecule (11). However, the functionality of this SNP and its role in affecting the sTREM-1 levels during active diseases are still inconsistent (32–35). As TREM-1 is a key effector of innate immunity, the presence of CC+CT genotypes associated with a lower expression of mbTREM-1 in the cells increases the odds of developing the disease.

Conversely, our functional data also showed that the TT genotype is related to higher production of sTREM-1, which is associated with clinical complications of leprosy. Altogether, our findings suggest that the TREM-1 SNP may affect the risk of leprosy occurrence, making this an important candidate gene for future studies in more powerful genetic studies. Conversely, the low producers' genotypes are associated with the infection. Moreover, the high inflammatory response associated with clinical outcomes in LR and PD patients is associated with the high production of sTREM-1 in leprosy patients, indicating the importance of a balanced immune response in leprosy.

This study has some limitations that need to be mentioned. All samples were collected and analyzed before the patient's treatment, and we considered only the occurrence of LR and PD at that moment. Notwithstanding, a prospective study evaluating serum-sTREM-1 and other biomarkers before clinical complications is required to assess if these biomarkers can predict these complications. Clearly, the future of biomarkers in leprosy diagnosis requires extensive validation studies of novel biomarkers across heterogeneous groups and evaluation of their power in combination with clinical and laboratory criteria. Moreover, our sample is limited to a small number of patients; thus, new investigations with more participants are required.

In light of the above, our main data showed that higher sTREM-1 levels helped us differentiate multibacillary patients from paucibacillary ones. These data also suggest that this molecule plays an important role in the pathogenesis of the inflammatory response in leprosy and provide a possible novel biomarker to assist in the diagnosis of leprosy's complications and their follow-up, although the mechanism whereby TREM-1 affects the initiation and progression of leprosy warrants further studies.

## Data availability statement

The raw data supporting the conclusions of this article will be made available by the authors, without undue reservation.

## Ethics statement

The studies involving human participants were reviewed and approved by Comitê de Ética em Pesquisa da Universidade Federal de Sergipe. Written informed consent to participate in this study was provided by the participants' legal guardian/next of kin.

## Author contributions

Concept and design: MB-S, SR, MD, RA, TM, and AJ. Acquisition of data: MB-S, LB, CS, MC, EM, LM, AB, MT, JA, LM-S, and RC. Analysis and interpretation of data: MB-S, LB, CS, MC, LM, AB, LM-S, RC, TM, and AJ. Drafting of the manuscript: MB-S, LB, CS, MT, JA, TM, and AJ. Statistical analysis: MB-S, CS, LM-S, RA, TM, and AJ. Obtained funding: SR, MD, ML, TM, and AJ. Critical revision of the manuscript for important intellectual content and final approval of manuscript: All authors.
