# Supplementary material for: sTREM-1 and TNF-α levels are associated with the clinical outcome of leprosy patients
Source: Front Med (Lausanne). 2023 Jun 29;10:1177375. doi: 10.3389/fmed.2023.1177375 (PMC10339318; doi:10.3389/fmed.2023.1177375)
Supplement: Supplementary file 1 [file Data_Sheet_1.pdf]

Supplementary Material

sTREM-1 and TNF-α levels are associated with the clinical outcome of leprosy patients

Márcio Bezerra-Santos, Lays G. Santos Bomfim, Camilla N. Oliveira Santos, Maria Wiliane N. Cunha, Eduardo J. Rocha de Moraes, Rodrigo A. Cazzaniga, Martha D. L. Tenório, Jonnia M. Sherlock Araujo. Lucas Menezes-Silva. Lucas Sousa Magalhães, Aline S. Barreto, Steven G. Reed, Malcolm S. Duthie, Michael W. Lipscomb, Roque Pacheco de Almeida, Tatiana Rodrigues de Moura*, Amélia Ribeiro de Jesus

*** Correspondence:** Tatiana Rodrigues de Moura: tmoura.ufs@gmail.com

Supplementary Table 1 Identification and characterization of genotyped single nucleotide polymorphisms

Gene	SNP	Chromosome / Location	SNP type	Allele	SNP *Assay*
TREM-1	rs2234246	Chr 6/ 3'UTR	Transition substitution	C>T	C___1165058_20
TNFα	rs1800629	Chr 6/ Intragenic	Transition substitution	G>A	C___7514879_10

Supplementary Table 2 Demographic and clinical characteristics of leprosy patients according to the serum-sTREM-1 levels

Variables	sTREM-1 >210 pg/ml n (%)	Odds Ratio	*p*-value	95% CI
Male	11 (55%)	1.49	0.56	0.47 to 4.68
MB form	15 (65.22%)	2.81	0.09	0.86 to 9.09
LL clinical form	07 (63.63%)	4.81	0.04	0.89 to 25.8
Leprosy Reaction	13 (54.16%)	2.81	0.06	0.88 to 8.88
Physical Disability	15 (62.5%)	5.83	0.004	1.71 to 19.91
MB + Leprosy Reaction	11 (76.92%)	1.83	0.47	0.30 to 8.18
PB + Leprosy Reaction	02 (25%)	2.16	0.48	0.27 to 15.86

Herein, we grouped the leprosy patients into those with serum-sTREM-1 levels >210 pg/ml and ≤210 pg/ml. Data were analyzed by Fisher exact test.
Abbreviations: MB, Multibacillary form; PB, Paucibacillary form; LL, Lepromatous Leprosy; CI, Confidence Interval.

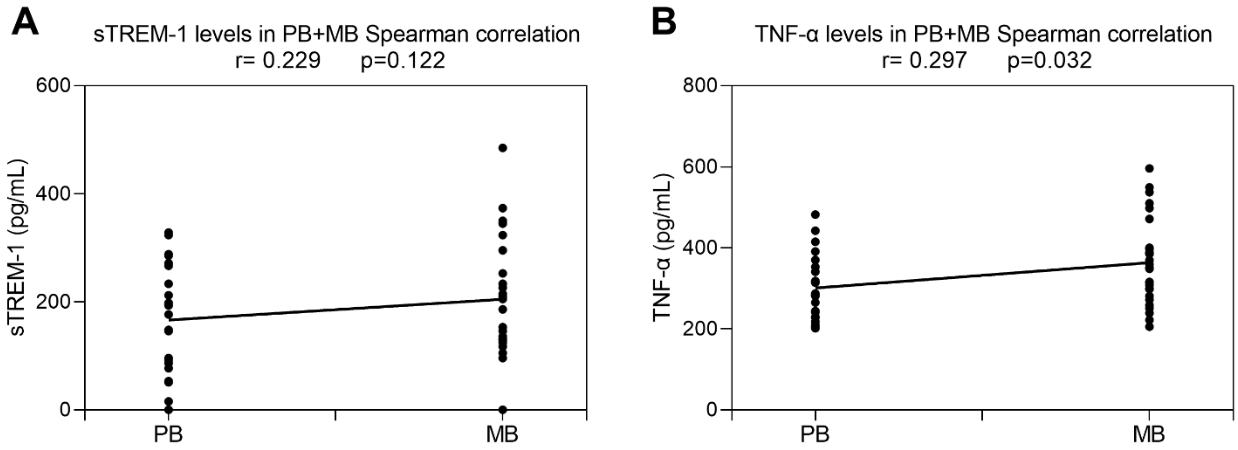

Supplementary Figure 1. Correlation among Paucibacilary and Multibacilary classification and sTREM-1 and TNF-α levels. A) sTREM-1 serum levels according to the operational classification on leprosy: multibacillary (MB) and paucibacillary (PB). **B)** TNF-α serum levels according to the operational classification on leprosy: multibacillary (MB) and paucibacillary (PB). Data was analyzed using point-biserial correlation comparing continuous values of cytokines quantification and dichotomous distribution between PB and MB groups.

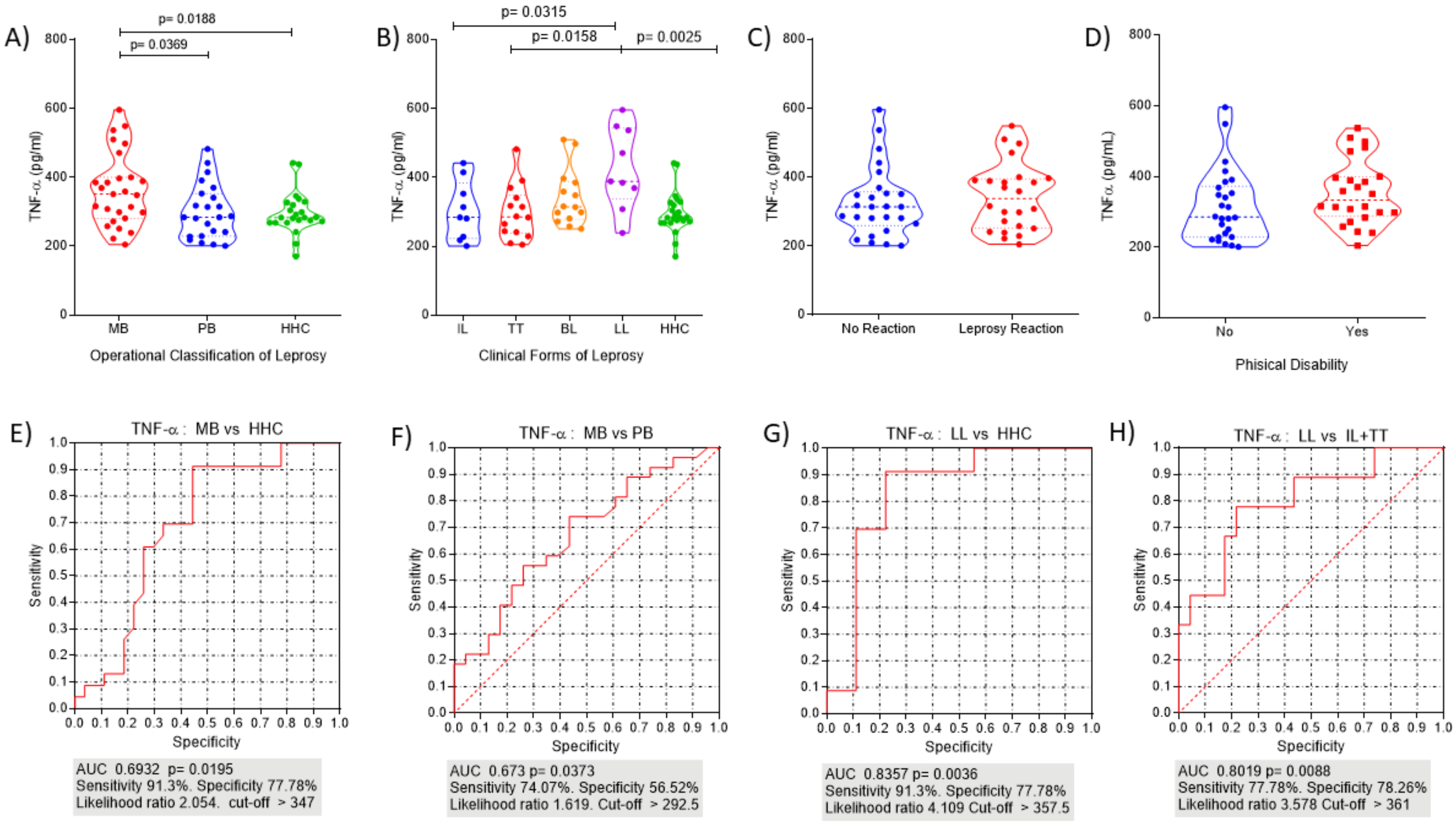

Supplementary Figure 2. TNF-α serum levels are associated to the MB presentation and the LL clinical form on leprosy. A) TNF-α serum levels according to the operational classification on leprosy: multibacillary (MB - in red), paucibacillary (PB - in blue) and household contacts (HHC - in green). **B)** TNF-α serum levels according to the clinical forms of leprosy: indeterminate (IL – in blue), tuberculoid (TT – in red), borderline (BL – in orange), lepromatous (LL – in violet) and HHC (in green). **C)** TNF-α serum levels according to the occurrence of leprosy reaction (LR): no reaction (in blue) and LR (in red). **D)** TNF-α serum levels according to the occurrence of physical disability (PD): no PD (in blue) and with PD (in red). Receiver operating characteristic (ROC) curve was generated for discriminates the levels of TNF-α between: **E)** leprosy patients presenting the MB operational form from HHC (area under the ROC curve [AUC] = 0.6932; *p*-value = 0.01); **F)** leprosy patients presenting the MB operational form from PB (AUC = 0.673; *p*-value = 0.03); **G)** leprosy patients presenting the LL clinical form from HHC (AUC = 0.8357; *p*-value = 0.003); **H)** leprosy patients presenting the LL clinical form from IL+TT (AUC = 0.8019; *p*-value = 0.008).

Supplementary Table 3 Demographic characteristics of patients and controls

Variables	LP (n = 210)	HHC (n =53)	EC (n= 115)	*p*-value
**Age**				
(Variation)	6 – 88	20 - 80	17 - 46	*<0.0001
Mean ± SD	45.93 ± 18.4	47.02 ± 12.2	22.4 ± 4.7	
**Male** n (%)	109 (51.9%)	16 (30.2%)	49 (42.6%)	**0.120
	**IL (n = 19)**			
**Age**				
(Variation)	8 - 81	---	---	---
Mean ± SD	39.5 ± 16.4			
**N. of lesions**				
(Variation)	1 - 5	---		---
Mean ± SD	2.9 ± 1.48		---	
	**TT (n = 58)**			
**Age**				
(Variation)	10 - 80	---	---	---
Mean ± SD	45.3 ± 18			
**N. of lesions**				
(Variation)	1 - 20	---		---
Mean ± SD	2.7 ± 3.58		---	
	**BL (n = 40)**			
**Age**				
(Variation)	6 - 88	---	---	---
Mean ± SD	48.8 ± 20.8			
**N. of lesions**				
(Variation)	1 - 15	---		---
Mean ± SD	5.7 ± 2.92		---	
	**LL (n = 52)**			
**Age**				
(Variation)	10 - 83	---	---	---
Mean ± SD	46.1 ± 18			
**N. of lesions**				
(Variation)	1 - 40	---		---
Mean ± SD	9.06 ± 8.1		---	

Abbreviations: LP, Leprosy patients; HHC, Household contacts; EC, Endemic controls; IL, Indeterminate leprosy; TT, Tuberculoid leprosy; BL, Borderline leprosy; LL, Lepromatous leprosy; SD, Standard deviation;

[a] Percentual of male subjects.

*Unpaired test; X^2, Chi-squared value; **Chi-squared test performed.

Supplementary Table 4 Allelic distribution of TREM-1 rs2234246 and TNFα rs1800629 compared by severity of leprosy

Allele frequencies n (%)[a]						
		Case group	Allele/ Genotype	OR	95% CI	*p*[b]
TREM-1 rs2234246	PB *n* = 75	MB *n* = 95				
C<br>T	71 (47.33)<br>79 (52.67)	80 (42.1)<br>110 (57.9)	C vs. T	0.809	0.5228 – 1.250	0.379
TNFα rs1800629	PB *n* = 73	MB *n* = 120				
A<br>G	17 (11.64)<br>129 (88.36)	21 (8.75)<br>219 (91.25)	A vs. G	0.727	0.3792 -1.463	0.381
TREM-1 rs2234246	Leprosy Reaction *n* = 62	No Leprosy Reaction *n* = 33				
C<br>T	55 (44.4)<br>69 (55.6)	27 (40.9)<br>39 (59.1)	C vs. T	0.868	0.4829 – 1.601	0.758
TNFα rs1800629	Leprosy Reaction *n* = 71	No Leprosy Reaction *n* = 33				
A<br>G	12 (8.4)<br>130 (91.6)	10 (15.2)<br>56 (84.8)	A vs. G	0.516	0.2227 – 1.289	0.152
TREM-1 rs2234246	PD Degree 1 or 2 *n* = 88	PD Degree 0 *n* = 48				
C<br>T	79 (44.9)<br>97 (55.1)	40 (41.7)<br>56 (58.3)	C vs. T	1.140	0.6994 – 1.887	0.701
TNFα rs1800629	PD Degree 1 or 2 *n* = 93	PD Degree 0 *n* = 53				
A<br>G	14(7.5)<br>172 (92.5)	13 (12.3)<br>93 (87.7)	A vs. G	1.717	0.7842 – 3.672	0.208

Abbreviations: HHC, household contacts; PB, paucibacillary leprosy; MB, Multibacillary leprosy, PD, Physical disability.

Abbreviations: OR, odds ratio; CI, confidence interval;

[a] (%) percentual of the subjects with the specified allele.

[b] Test for association were performed using Fisher exact test

Supplementary Table 5 Allelic distribution of TREM-1 rs2234246 and TNFα rs1800629 compared by clinical forms of leprosy

	Allele frequencies n(%)[a] Case group				Allele/Genotype	X^2	p^b
TREM-1 rs2234246 *n=153*	IL *n* = 21	TT *n* = 52	BL *n* = 45	LL *n* = 35			
C	21 (50)	50 (48.1)	36 (40)	31 (44.3)	T vs. C	1.744,3	0.627
T	21 (50)	54 (51.9)	54 (60)	39 (55.7)			
TNFα rs1800629 *n=169*	IL *n* = 19	TT *n* = 58	BL *n* = 40	LL *n* = 52			
A	5 (13.2)	14 (12)	5 (6.25)	9 (8.7)	A vs G	2.463, 3	0.482
G	33 (86.8)	102 (88)	75 (93.75)	95 (91.3)			

Abbreviations: IL; Indeterminate leprosy; TT, Tuberculoid leprosy; BL, Borderline leprosy; LL, Lepromatous leprosy.
[a] (%) percentual of the subjects with the specified allele.
[b] Test for association were performed using Chi-squared test. Confidence interval 95%.

Supplementary Table 6 Association of higher producer sTREM-1 for leprosy patients and controls together

**Genotype frequencies** n (%)[a]		**Higher producer >210 pg/ml (n = 17)**	**Lower producer ≤210 pg/ml (n = 29)**	***p*-value [b]**
**TREM-1 rs2234246**	CC	3 (17.6)	3 (10.3)	
	CT	5 (29.4)	20 (69.0)	**0.03**
	TT	9 (53.0)	6 (20.7)	

[a] (%) percentage of the subjects with the specified genotype
[b] Test for association were performed using Chi-squared test

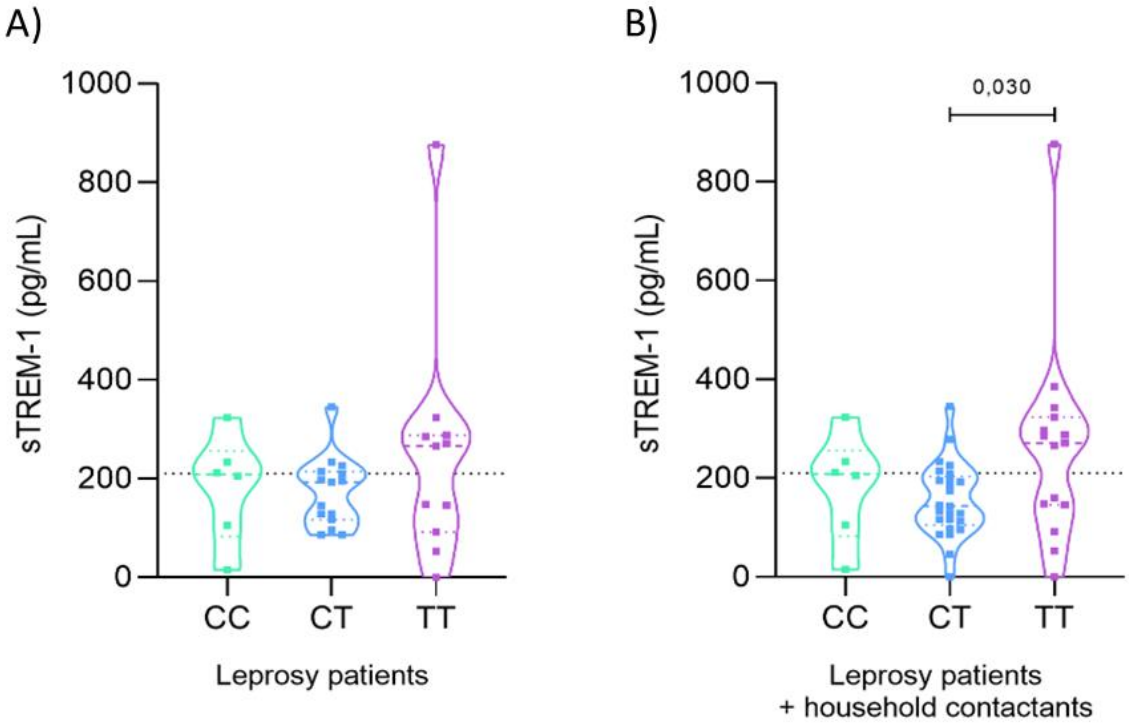

Supplementary Figure 3. Levels of sTREM-1 of leprosy patients and household controls distributed by genotypes for TREM-1 rs2234246. A) Levels of sTREM-1 in leprosy patients distributed by genotypes for TREM-1 rs2234246. **B)** Levels of sTREM-1 in leprosy patient + household control distributed by genotypes for TREM-1 rs2234246. CC, homozygous for the major allele of the rs2234246 (in green); CT, heterozygous for the rs2234246 (in blue); TT, homozygous for the minor allele of the rs2234246 (in purple). The dashed line indicates serum sTREM-1 levels above 210 pg/Ml. The significance between genotypes was performed by Mann-Whitney test (*p*-value <0.05).
